# AFM Probing the Mechanism of Synergistic Effects of the Green Tea Polyphenol (−)-Epigallocatechin-3-Gallate (EGCG) with Cefotaxime against Extended-Spectrum Beta-Lactamase (ESBL)-Producing *Escherichia coli*

**DOI:** 10.1371/journal.pone.0048880

**Published:** 2012-11-13

**Authors:** Yidan Cui, So Hyun Kim, Hyunseok Kim, Jinki Yeom, Kisung Ko, Woojun Park, Sungsu Park

**Affiliations:** 1 Department of Chemistry and Nano Sciences, Ewha Womans University, Seoul, Korea; 2 Division of Environmental Science and Ecological Engineering, Korea University, Seoul, Korea; 3 Mechanobiology Institute, National University of Singapore, Singapore, Singapore; 4 Department of Medicine, College of Medicine, Chung-Ang University, Seoul, Korea; University of South Florida College of Medicine, United States of America

## Abstract

**Background:**

Extended-spectrum β-lactamase (ESBL)-producing Enterobacteriaceae poses serious challenges to clinicians because of its resistance to many classes of antibiotics.

**Methods and Findings:**

The mechanism of synergistic activity of a combination of (−)-epigallocatechin-3-gallate (EGCG) and β-lactam antibiotics cefotaxime was studied on Extended-spectrum β-lactamase producing *Escherichia coli* (ESBL-EC), by visualizing the morphological alteration on the cell wall induced by the combination using atomic force microscopy (AFM). Cells at sub-MICs (sub-minimum inhibitory concentrations) of cefotaxime were initially filamentated but recovered to the normal shape later, whereas cells at sub-MICs of EGCG experienced temporal disturbance on the cell wall such as leakage and release of cellular debris and groove formation, but later recovered to the normal shape. In contrast, the combination of cefotaxime and EGCG at their respective sub-MICs induced permanent cellular damages as well as continuous elongation in cells and eventually killed them. Flow cytometry showed that intracellular oxidative stress levels in the cell treated with a combination of EGCG and cefotaxime at sub-MICs were higher than those in the cells treated with either cefotaxime or EGCG at sub-MICs.

**Conclusions:**

These results suggest that the synergistic effect of EGCG between EGCG and cefotaxime against ESBL-EC is related to cooperative activity of exogenous and endogenous reactive oxygen species (ROS) generated by EGCG and cefotaxime, respectively.

## Introduction

The reemergence of infectious diseases and the continuous development of antimicrobial drug resistance in pathogens have been causing an alarming deficit in effective antibacterial agents, leading to a growing threat to public healthcare worldwide. Extended-spectrum β-lactamase (ESBL)-producing Enterobacteriaceae have become the most frequent nosocomial pathogens and have posed serious challenges to clinicians because of their resistance to many classes of antibiotics [1]. ESBLs are the enzymes produced by Gram-negative bacteria that mediate resistance to third-generation cephalosporins (such as cefotaxime and ceftriaxone) by hydrolysis of these antibiotics [2]. Plasmid-mediated ESBL enzymes are of special interest in the generation of ESBL variants [3]. Infections caused by Enterobacteriaceae producing ESBL often complicate the therapy and limit treatment options, often necessitating combination therapy [4]. Combinations of a β-lactam with either a β-lactamase inhibitor or a fluoroquinolone, or double β-lactam combinations are common, but these may not always prevent the emergence of resistance [4], [5]. Several reviews recently emphasized the urgent need for new therapeutic strategies [6], [7].

(−)-Epigallocatechin-3-gallate (EGCG), a main constituent of green tea polyphenol, has been reported to have great anti-infective potential [8], [9] and also aids other antibiotics against both antibiotic-resistant Gram-positive [10], [11] and Gram-negative bacteria [12], [13] at sub-minimum inhibitory concentrations (sub-MICs). Synergy between EGCG and β-lactam against β-lactamase producing *Staphylococcus aureus* can be easily explained by the fact that both antibacterial compounds attack the same site of the peptidoglycan layer in Gram-positive bacteria [11], [14]. Since EGCG is not able to bind the peptidoglycan layer of Gram-negative bacteria because of their outer membrane, it is suggested that the mechanism underlying the synergistic effect of EGCG with the antibiotics against Gram-negative bacteria is different from the mechanism underlying the synergistic effects against Gram-positive bacteria.

Atomic force microscopy (AFM) is a very useful tool for visualizing the morphology of bacterial surfaces in nanoscale, and has been used to study the antibacterial effects of antibiotics [15], [16], antimicrobial peptides [17], [18], and others [19], [20]. It has been most recently shown by AFM that EGCG has different modes of antibacterial action against Gram-negative and Gram-positive bacteria by direct binding to the peptidoglycan layer or through H_2_O_2_ production, respectively [19].

In this study, we used AFM to obtain high-resolution images of morphological changes in ESBL-*Escherichia coli* induced by a sole treatment of EGCG or cefotaxime at sub-MICs (sub-minimum inhibitory concentrations) or a co-treatment of EGCG and cefotaxime at their respective sub-MICs. To explain the cause of the morphological changes on the bacterial cell surface, oxidative stress response in ESBL-EC against the treatments were measured.

## Materials and Methods

### Bacterial Strain and Growth Condition

An ESBL-EC strain (BAA-198) [21] was obtained from ATCC. The strain was grown overnight with aeration in a round glass tube containing Mueller – Hinton Broth (MHB; not cation-adjusted; Becton Dickinson) at 37°C. The overnight culture was 100 times diluted with MHB to a final volumes of 5 ml and continued growing with aeration at 37°C till reaching stationary phase determined from optical density at 600 nm (OD_600_ = 4). Then those cultures with OD_600_ value of 4 were 100 times diluted with MHB and grown with various concentrations of EGCG (Sigma-Aldrich, St. Louis, MO), cefotaxime (Beta-Lactam antibiotics; Sigma-Aldrich) or their combinations either for MIC determination or time-kill studies. Bacterial growth was calculated by colony-forming unit (CFU) count. CFUs were measured by counting colonies after plating 20 µl of each culture on MHB plates and incubating the plates overnight.

**Table 1:** MIC and FIC indices of cefotaxime in combination with EGCG against ESBL-EC.

	MIC (mg/L)				FIC Index^a^			Effect
	A	B	C	D	B	C	D	
**Cefotaxime**								
	128	8	4	4	0.1	0.1	0.2	Synergy^b^

A, Cefotaxime alone; B, plus EGCG (50 mg/L); C, plus EGCG (100 mg/L); D, plus EGCG (250 mg/L). The MIC of EGCG alone was 1500 mg/L.

a Fractional inhibitory concentration (FIC) was calculated as MIC of antibiotics alone or EGCG in com-bination divided by MIC of antibiotics or EGCG alone, and the FIC Index was obtained by adding theFICs.

b FIC indices were interpreted as below: ≤0.5, synergy; >0.5 to 1, addition; and >1, indifference.

### Determination of MIC and Confirmation of the Synergistic Effect

MICs were determined by broth microdilution method [11]. Tubes containing various concentrations of EGCG or cefotaxime (or both) in MHB media were prepared. The total volume of media in each tube was 5 ml. The media were then inoculated with 50 µl of *E. coli* suspensions (OD_600_ = 4) and incubated at 37°C with aeration for 18 h. MIC is defined as the lowest concentration of an antimicrobial agent at which no visible growth will occur after overnight incubation.

The effects of combinations were confirmed by the checkerboard method [22]. Two-fold serial dilutions of cefotaxime were tested in combinations with serial dilutions of EGCG. The results were evaluated by a fractional inhibitory concentration (FIC) index. FIC was calculated as MIC of antibiotics alone or EGCG in combination divided by MIC of antibiotics or EGCG alone, and the FIC index was obtained by adding the FICs. FIC indices were interpreted as follows: ≤0.5, synergy; >0.5 to 1, addition; and >1, indifference.

### Time-kill Studies

Time-related effects of EGCG, cefotaxime and their combinations were determined by measuring cultures’ CFUs. *E. coli* suspensions (OD_600_ = 4) were 100 times diluted with MHB media containing different concentrations of cefotaxime or EGCG (or both). The number of surviving bacteria was counted after 0, 1, 2, 4, 6, 8, 10, 12, 14, 16 and 18 h incubated at 37°C.

### Scanning Electron Microscopy (SEM) Analysis

Bacterial suspensions were pre- and post-fixed in glutaraldehyde solution and then added to glass cover slips. The glass cover slips were dehydrated in a series of ethanol concentrations (30–95%) for 15 min followed by 100% ethanol for 20 min. Then the cells were coated with gold and imaged by scanning electron microscopy.

### Preparation of AFM Samples

For AFM imaging in air, aliquots of bacterial suspension were added to glass cover slips and rinsed gently in deionized water. The glass cover slips were then dried in a covered Petri dish (50 mm × 50 mm) for several minutes and immediately moved to AFM for imaging.

### AFM Operation

The AFM images were taken in air with a commercial AFM (Dimension 3100 with a Nanoscope III controller, Digital Instruments). All images were collected in tapping mode using silicon cantilevers (RTESP, Veeco Probes) with resonance frequencies of approximately 300 kHz and spring constants of approximately 50 Nm^−1^. Cantilevers were not reused to prevent cross-contamination. Height and amplitude images were simultaneously acquired at scan rates of 0.5−1 Hz with 512×512 resolutions. All visible images were given as amplitude images. To describe the topography of the bacterial surface quantitatively, section analyses and root-mean-square roughnesses (R_rms_) were taken from the height images. Once AFM images of single cells were acquired, we selectively magnified areas (500 nm × 500 nm) of the cells except their both ends. Then, we randomly selected very small regions (500 nm^2^) from the magnified areas to reduce the effect of bacterial half cylindrical structure on R_rms_ calculation. Membrane roughness was an average of these values taken from at least 20 cells.

### Oxidative Stress Detection using Flow Cytometry

Dihydrorhodamine 123 (DHR 123, Sigma), an oxidative stress probe, was used to measure intracellular oxidation levels in bacteria [23] after chemical treatment. EGCG, cefotaxime, or combinations thereof were added to the culture and incubated for either 4 h or 8 h. Then, cells were washed in phosphate-buffered saline (PBS) and incubated with 5 mg/L of DHR 123 for 1 h. Finally, the cells were washed, resuspended in PBS and transferred to flow cytometry tubes. The fluorescence intensity of the bacteria was measured by a FACSCalibur™ flow cytometer (BD), and the mean fluorescence channel (MFC) value of 20,000 cells was determined upon analysis of the live or dead cell population, which was defined by forward and side scatter using CellQuest Pro software (BD). Data were analyzed in FSC/FL-1 histograms.

## Results

### Synergistic Effect of Cefotaxime and EGCG Against ESBL-EC

MICs of cefotaxime and EGCG were determined to be 128 mg/L and 1500 mg/L, respectively. Any combinations of cefotaxime and EGCG at their sub-MICs showed synergistic effect against ESBL-EC (Table 1). Cefotaxime at 8 mg/L showed synergistic effect with the lowest concentration (50 mg/L) of EGCG, while EGCG at both 100 mg/L and 250 mg/L showed synergistic effect with the lowest concentration (4 mg/L) of cefotaxime.

### Time Dependent Effects of Cefotaxime and EGCG Against ESBL-EC

**Figure 1:**
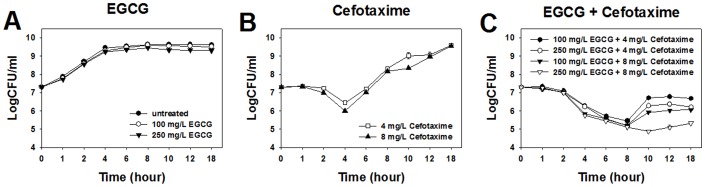
Time-kill curves of ESBL-EC treated with EGCG and cefotaxime at sub-MICs.

The bacterial growth was not affected by the treatment of EGCG at either 1/15 MIC (100 mg/L) or 1/6 MIC (250 mg/L) (Figure 1A), while it was inhibited up to 4 h after the treatment of cefotaxime at either 1/32 MIC (4 mg/L) or 1/16 MIC (8 mg/L) (Figure 1A and 1B). It was inhibited for more than 8 h at co-treatment of cefotaxime and EGCG at their respective sub-MICs (Figure 1C). Especially when co-treated with cefotaxime and EGCG at their respective 1/16 MIC and 1/6 MIC, bacterial growth was inhibited for up to 10 h (Figure 1C). These results indicate that the inhibitory effect of cefotaxime against ESBL-EC was extended by the addition of EGCG.

### SEM Observation of Morphological Changes in ESBL-EC Induced by Cefotaxime and EGCG

**Figure 2:**
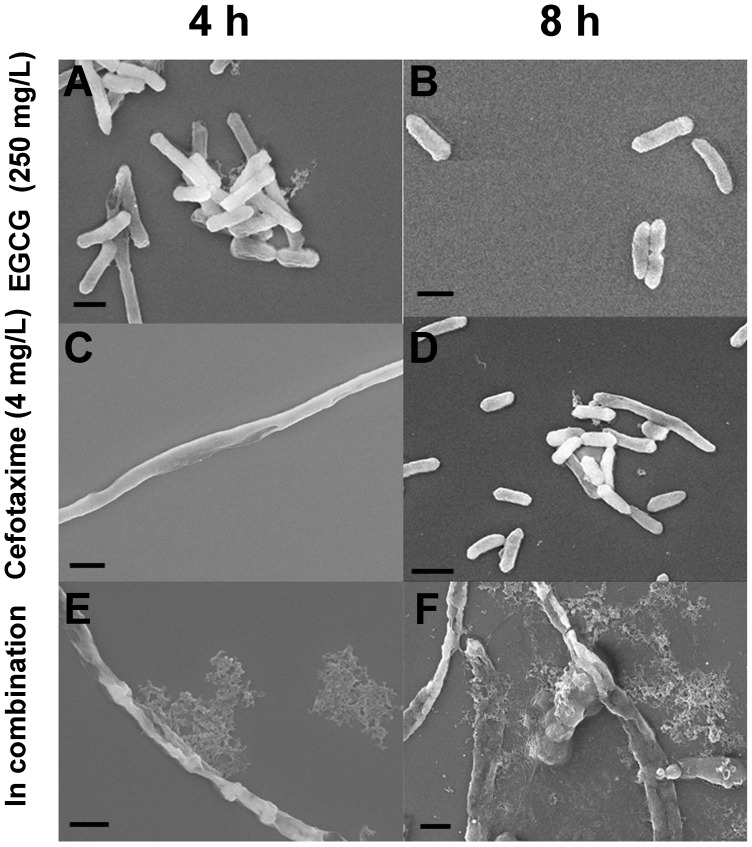
SEM images of ESBL-EC treated with sub-MICs of EGCG, cefotaxime or their combinations. Cells were: treated with 250 mg/L of EGCG for 4 h (A) and 8 h (B); treated with 4 mg/L of cefotaxime for 4 h (C) and 8 h (D); and treated with 250 mg/L of EGCG and 4 mg/L of cefotaxime in combination for 4 h (E) and 8 h (F). Scale bar: 1 µm.

When treated with EGCG at 1/6 MIC (250 mg/L) for 4 h and 8 h, neither morphological changes nor significant leakages were observed in the cells (Figure 2A and 2B). When treated with cefotaxime at 1/32 MIC (4 mg/L), cells were elongated at 4 h (Figure 2C), but the normal cell shape was restored at 8 h (Figure 2D). When co-treated with cefotaxime at 1/32 MIC and EGCG at 1/6 MIC, cells were elongated and lost their cellular contents, leaving debris around them at 4 h (Figure 2E). However, they were not able to restore their normal cell shape at 8 h and became more severely damaged (Figure 2F).

### AFM Observation of Morphological Changes in *ESBL*-EC Induced by Cefotaxime and EGCG

**Figure 3:**
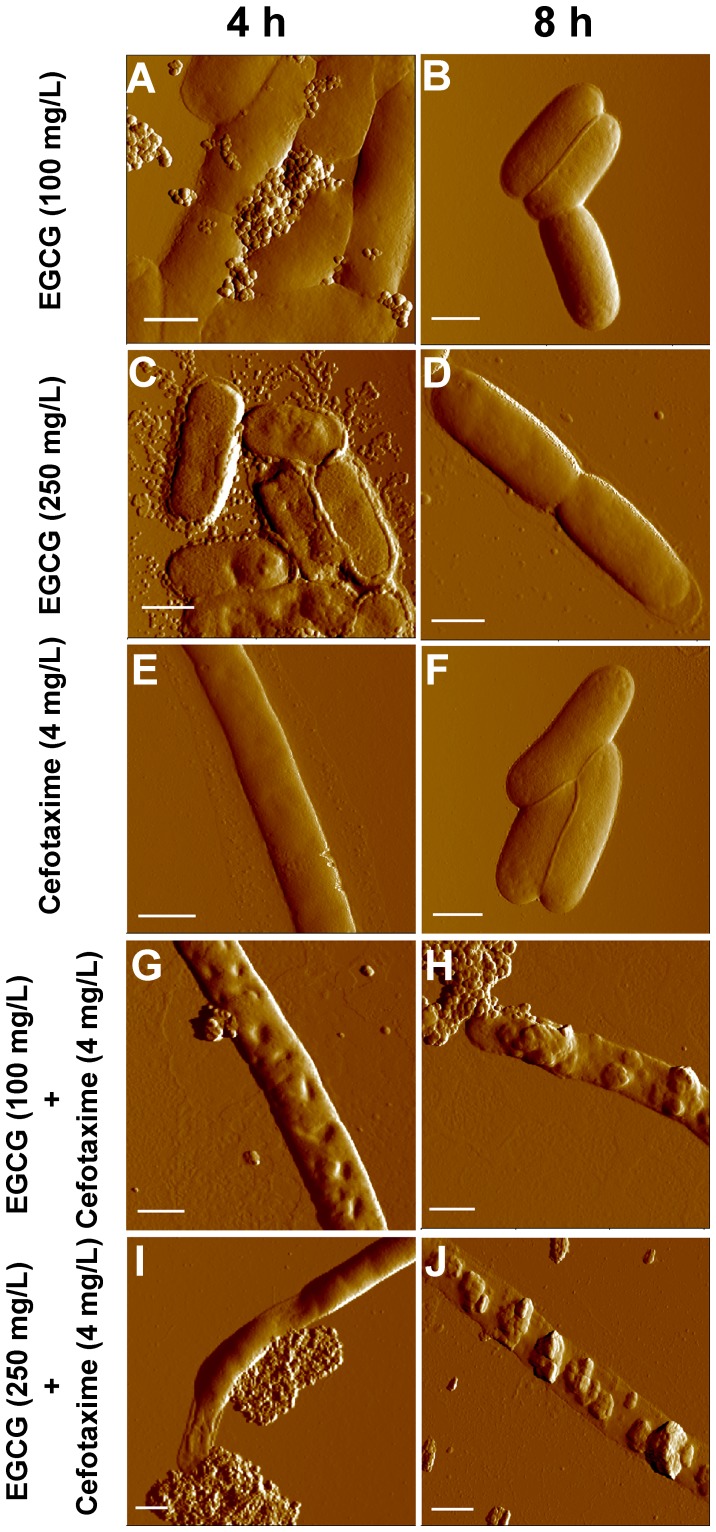
Topological images of ESBL-EC treated with sub-MICs of EGCG, cefotaxime or their combinations. Cells were: treated with 100 mg/L of EGCG for 4 h (A) and 8 h (B); treated with 250 mg/L of EGCG for 4 h (C) and 8 h (D); treated with 4 mg/L of cefotaxime for 4 h (E) and 8 h (F); treated with 100 mg/L of EGCG and 4 mg/L of cefotaxime in combination for 4 h (G) and 8 h (H); and treated with 250 mg/L of EGCG and 4 mg/L of cefotaxime in combination for 4 h (I) and 8 h (J). Scale bar: 1 µm.

Untreated *E. coli* cells have very smooth surfaces (R_rms_ 1.3±0.1 nm, *n* = 20) (Figure S1). When treated with EGCG at 100 mg/L for 4 h, some leakages (Figure 3A) were observed in cells with slightly increased roughness (R_rms_ 6.8±1.8 nm, *n* = 20), possibly due to the leakages. Cells restored their smooth surface (Figure 3B) at 8 h and their surface displayed a low roughness (R_rms_ 1.5±0.6 nm, *n* = 20). When treated with EGCG at 250 mg/L for 4 h, large grooves were observed over the cell wall, leading to rough cell surfaces (R_rms_ 14.9±1.0 nm, *n* = 20) (Figure 3C). Occasionally, partial collapse of the cell wall was also observed and more debris was released from cell, indicating that cells were more damaged at 250 mg/L than at 100 mg/L. Like at 100 mg/L of EGCG, *E. coli* at 250 mg/L of EGCG also recovered from such damages at 8 h (R_rms_ 2.4±0.6 nm, *n* = 20) (Figure 3D). The treatment of cefotaxime at 4 mg/L for 4 h mostly induced elongation (Figure 3E). Interestingly, elongated cells were able to maintain relatively smooth surfaces (R_rms_ 3.6±1.0 nm, *n* = 20) on their cell walls (Figure 3E). With the same treatment, some cells were not elongated, but their surface was uneven as shown in supporting information (Figure S2B), and some other cells showed leakages with flattened cell bodies (Figure S2C). At 8 h, cells restored their native rod shape (Figure 3F) with smooth surfaces (R_rms_ 1.4±0.5 nm, *n* = 20).

When co-treated with cefotaxime (4 mg/L) and EGCG (100 mg/L), cells became elongated with large grooves and partial leakages on their cell walls at 4 h (Figure 3G), increasing their surface roughness (R_rms_ 22.0±9.5 nm, *n* = 20). At 8 h, more severe leakages were observed all along the cell body, and the damaged cells lost their cellular contents, leaving flattened cell bodies and extremely rough cell surfaces (R_rms_ 43.2±12.6 nm, *n* = 20; Figure 3H). When treated with both cefotaxime (4 mg/L) and EGCG (250 mg/L), the leakage of vast cellular materials resulted in the partial flattening of cell bodies as well as increase in surface roughness (R_rms_ 40.0±7.3 nm, 21 *n* = 20) at 4 h (Figure 3I). At 8 h, the cell walls were totally collapsed, and severe cellular leakage caused flattening of the cells, leaving only empty envelopes (Figure 3J) and the highest roughness (R_rms_ 76.0±14.1 nm, *n* = 20). AFM images of whole cells for each co-treatment can be found in the Supporting Information section (Figure S3A – S3D).

### Oxidative Stress Level in ESBL-EC Treated with Cefotaxime and EGCG

**Figure 4:**
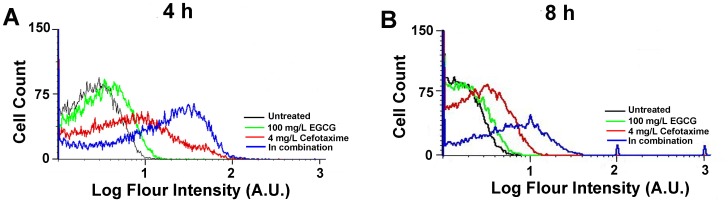
Oxidative stress response in ESBL-EC treated with sub-MICs of EGCG, cefotaxime or their combinations. Cells were either treated for 4 h (A) or 8 h (B).

DHR was used as an indicator of intracellular H_2_O_2_ [23]–[26]. At both 4 h and 8 h, bacterial cells co-treated with EGCG and cefotaxime showed higher oxidative stress response than those treated with either EGCG or cefotaxime (Figure 4A and 4B). The cells with any of the treatments for 4 h showed higher oxidative stress response than those at 8 h (Figure 4A and 4B), indicating that antibacterial effects of all the treatment decrease over time.

## Discussion

The sole treatment of either cefotaxime or EGCG at sub-MICs temporarily inhibits ESBL-EC. The cefotaxime induced filamentation in the cells. Filamentation is a typical feature of the morphological change induced by β-lactam antibiotics against Gram-negative bacteria [15], [27]. It was reported that β-lactam antibiotics induced SOS response in *E. coli.* During the SOS response, bacteria stop dividing and become filamentated. As a result, filamentated bacteria were able to survive under the selective pressure of β-lactam antibiotics [2], [27]. Similarly, filamenated cells were observed at 4 h after cefotaxime treatment, as shown in Figure 2C and 3E. Neither damages nor leakage were observed in the filamentated cells (Figure 3E and S2A), indicating that the cell walls maintained integrity during filamentation. ESBL-EC can obtain resistance to cefotaxime at 8 h. The strain produces β-lactamase to hydrolyze β-lactam antibiotics [2]. This might cause the strain to recover normal cell shape (Figure 2D and 3F) as well as normal growth rate (Figure 1B). Although EGCG at sub-MICS induced cellular damages in ESBL-EC (Figure 3A and 3C), their growth was not significantly inhibited by the treatments (Figure 1A). Furthermore, the strain recovered normal shape at 8 h (Figure 3B and 3D), suggesting that EGCG can cause only a temporal disturbance on the cell wall of ESBL-EC. EGCG is known to generate reactive oxygen species (ROS) by auto-oxidation [28], [29]. The production rate of H_2_O_2_ by EGCG increases greatly in the first hour and decreases thereafter [28].

ESBL-EC was not able to withstand oxidative stress exerted by co-treatment of EGCG and cefotaxime. Similarly, its growth was more severely inhibited by co-treatment of H_2_O_2_ and cefotaxime, compared to in the sole treatment of either H_2_O_2_ or cefotaxime (Figure S4C). During the SOS response against oxidative stress, cells become filamentated until they remove oxidative stress agents such as H_2_O_2_ and antibiotics. Since ESBL-EC cells were kept filamentated with severe damages even at 8 h (Figure 3H and 3J) by the co-treatment, it is suggested that ESBL-EC cells are not able to hydrolyze cefotaxime in the presence of EGCG. This can lead to a hypothesis that β-lactamase is damaged by excessive oxidative stress induced by the co-treatment. As shown in Figure 4, in fact, the cells upon the co-treatment experienced a higher oxidative stress than those upon the sole treatment of either EGCG or cefotaxime. Gram-negative bacteria experience oxidative stress due to H_2_O_2_ produced by EGCG [19], [28]. The inhibitory effect of β-lactam antibiotics is also known to be related to endogenous hydroxyl radical (OH**^•^**) damage, which initiates SOS response [30]. The cell wall of Gram-negative bacteria is not likely to be directly attacked by EGCG due to the presence of the outer membrane and lipopolysaccharide. Herein, we propose a mechanism for the synergistic effect between cefotaxime and EGCG as a converging attack of exogenous and endogenous oxidative stress generated by EGCG and cefotaxime, respectively. Oxidative stress not only initiates membrane degradation, but also causes cell wall collapse and significantly disrupts cellular proteins [24]. Our AFM images and FACS data suggest that the synergistic effect between EGCG and cefotaxime thus may be explained as the synergy between exogenous and endogenous ROS, which are lethal to ESBL-EC. Similarly,

Synergistic effect between cefotaxime and EGCG was only observed when EGCG was used at 100 mg/L, which is considerably above physiological levels. Therefore, combined use of EGCG with cefotaxime could be useful only for topical application to the skin infected with ESBL-EC.

## Supporting Information

Figure S1 **Topological images of ESBL-EC without any antibacterial treatment.**
(DOCX)

Figure S2 **Topological images of elongated ESBL-EC and cells failed in filamentation.** Cells were: elongated (A); ghost cell (B) and severely leaked cell (C) after treatment of cefotaxime at 4 mg/L for 4 h.
(DOCX)

Figure S3 **Topological images of ESBL-EC co-treated with sub-MICs of EGCG and cefotaxime.** Cells were: treated with 100 mg/L of EGCG and 4 mg/L of cefotaxime in combination for 4 h (A) and 8 h (B) and treated with 250 mg/L of EGCG and 4 mg/L of cefotaxime in combination for 4 h (C) and 8 h (D). Scale bar: 10 µm.
(DOCX)

Figure S4 **Time-kill curves of ESBL-EC treated with H_2_O_2_ and cefotaxime at sub-MICs.**
(DOCX)
