# Supplementary figures and images for: AFM Probing the Mechanism of Synergistic Effects of the Green Tea Polyphenol (−)-Epigallocatechin-3-Gallate (EGCG) with Cefotaxime against Extended-Spectrum Beta-Lactamase (ESBL)-Producing Escherichia coli
Source: PLoS One. 2012 Nov 13;7(11):e48880. doi: 10.1371/journal.pone.0048880 (PMC3496731; doi:10.1371/journal.pone.0048880)

**
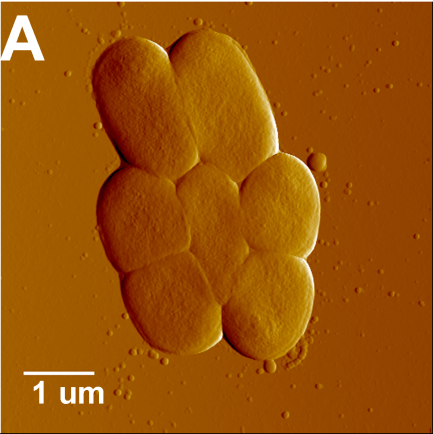
**

**Figure S1. Topological images of ESBL-ECwithout any antibacterial treatment.**

Supplement: Figure S1 — Topological images of ESBL-EC without any antibacterial treatment. (DOCX) [file pone.0048880.s001.docx]

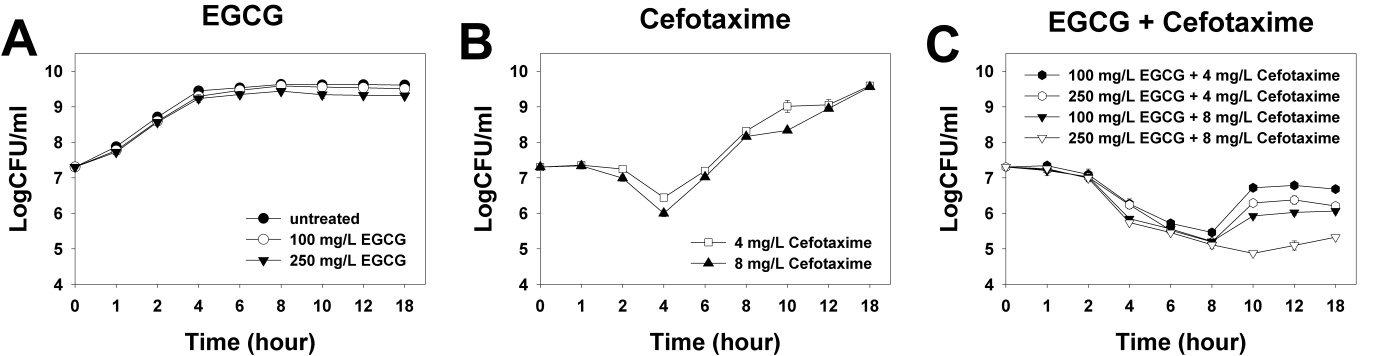


**Figure S4.** Time-kill curves of ESBL-EC treated with H2O2 and cefotaxime at sub-MICs.

Supplement: Figure S4 — Time-kill curves of ESBL-EC treated with H2O2 and cefotaxime at sub-MICs. (DOCX) [file pone.0048880.s004.docx]
