# Supplementary material for: AFM Probing the Mechanism of Synergistic Effects of the Green Tea Polyphenol (−)-Epigallocatechin-3-Gallate (EGCG) with Cefotaxime against Extended-Spectrum Beta-Lactamase (ESBL)-Producing Escherichia coli
Source: PLoS One. 2012 Nov 13;7(11):e48880. doi: 10.1371/journal.pone.0048880 (PMC3496731; doi:10.1371/journal.pone.0048880)
Supplement: Figure S2 — Topological images of elongated ESBL-EC and cells failed in filamentation. Cells were: elongated (A); ghost cell (B) and severely leaked cell (C) after treatment of cefotaxime at 4 mg/L for 4 h. (DOCX) [file pone.0048880.s002.docx]

**
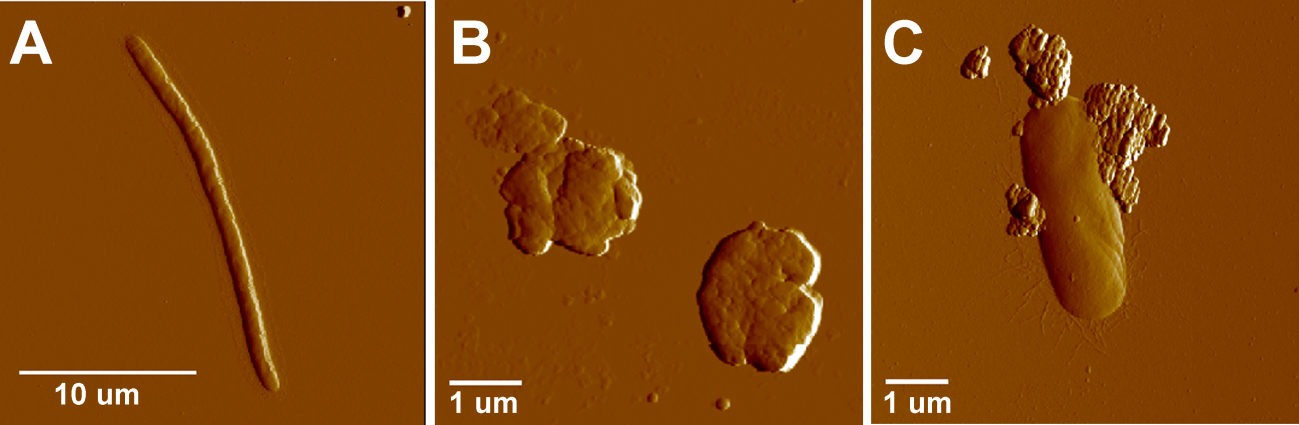
**

**Figure S2.** Topological images of elongated ESBL-EC and cells failed in filamentation. Cells were: elongated (A); ghost cell (B) and severely leaked cell (C) after treatment of cefotaxime at 4 mg/L for 4 h.
