# Supplementary material for: AFM Probing the Mechanism of Synergistic Effects of the Green Tea Polyphenol (−)-Epigallocatechin-3-Gallate (EGCG) with Cefotaxime against Extended-Spectrum Beta-Lactamase (ESBL)-Producing Escherichia coli
Source: PLoS One. 2012 Nov 13;7(11):e48880. doi: 10.1371/journal.pone.0048880 (PMC3496731; doi:10.1371/journal.pone.0048880)
Supplement: Figure S3 — Topological images of ESBL-EC co-treated with sub-MICs of EGCG and cefotaxime. Cells were: treated with 100 mg/L of EGCG and 4 mg/L of cefotaxime in combination for 4 h (A) and 8 h (B) and treated with 250 mg/L of EGCG and 4 mg/L of cefotaxime in combination for 4 h (C) and 8 h (D). Scale bar: 10 µm. (DOCX) [file pone.0048880.s003.docx]

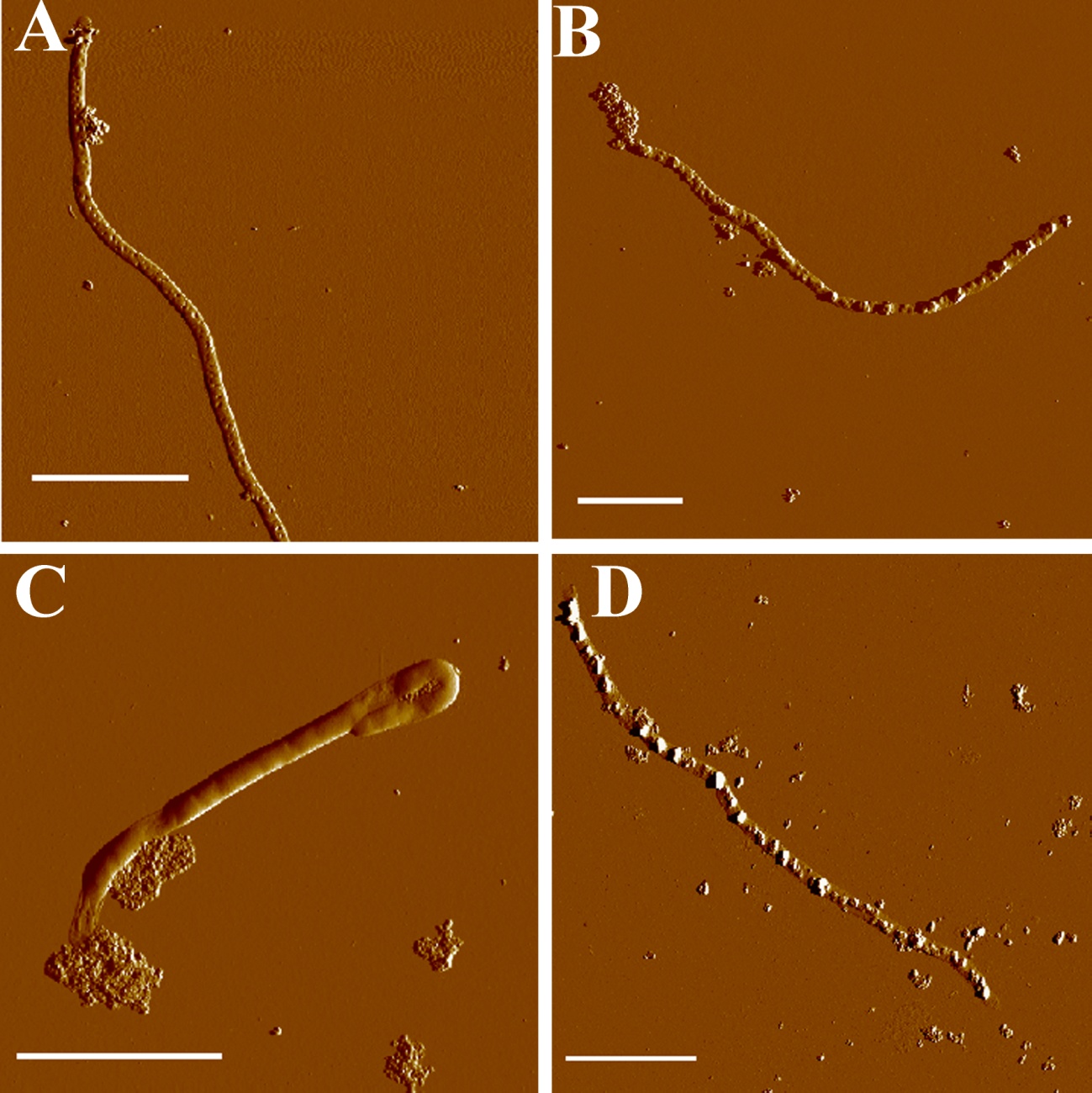


**Figure S3.** Topological images of ESBL-EC co-treated with sub-MICs of EGCG and cefotaxime. Cells were: treated with 100 mg/L of EGCG and 4 mg/L of cefotaxime in combination for 4 h (A) and 8 h (B) and treated with 250 mg/L of EGCG and 4 mg/L of cefotaxime in combination for 4 h (C) and 8 h (D). Scale bar: 10 µm.
